# Glioma Imaging by O-(2-18F-Fluoroethyl)-L-Tyrosine PET and Diffusion-Weighted MRI and Correlation With Molecular Phenotypes, Validated by PET/MR-Guided Biopsies

**DOI:** 10.3389/fonc.2021.743655

**Published:** 2021-11-29

**Authors:** Ye Cheng, Shuangshuang Song, Yukui Wei, Geng Xu, Yang An, Jie Ma, Hongwei Yang, Zhigang Qi, Xinru Xiao, Jie Bai, Lixin Xu, Zeliang Hu, Tingting Sun, Leiming Wang, Jie Lu, Qingtang Lin

**Affiliations:** ^1^ Department of Neurosurgery, Xuanwu Hospital, Capital Medical University, Beijing, China; ^2^ Department of Neurosurgery, China International Neuroscience Institute, Beijing, China; ^3^ Department of Neurosurgery, National Clinical Research Center for Geriatric Diseases, Beijing, China; ^4^ Department of Radiology and Nuclear Medicine, Xuanwu Hospital, Capital Medical University, Beijing, China; ^5^ Beijing Key Laboratory of Magnetic Resonance Imaging and Brain Informatics, Beijing, China; ^6^ Department of Nuclear Medicine, The Affiliated Hospital of Qingdao University, Qingdao, China; ^7^ Department of Pathology, Xuanwu Hospital, Capital Medical University, Beijing, China; ^8^ Department of Medicine, Nanjing Geneseeq Technology Inc., Nanjing, China

**Keywords:** hybrid PET/MR, ^18^F-FET, DWI, glioma phenotyping, biopsy

## Abstract

Gliomas exhibit high intra-tumoral histological and molecular heterogeneity. Introducing stereotactic biopsy, we achieved a superior molecular analysis of glioma using O-(2-18F-fluoroethyl)-L-tyrosine (FET)-positron emission tomography (PET) and diffusion-weighted magnetic resonance imaging (DWI). Patients underwent simultaneous DWI and FET-PET scans. Correlations between biopsy-derived tumor tissue values, such as the tumor-to-background ratio (TBR) and apparent diffusion coefficient (ADC)/exponential ADC (eADC) and histopathological diagnoses and those between relevant genes and TBR and ADC values were determined. Tumor regions with human telomerase reverse transcriptase (hTERT) mutation had higher TBR and lower ADC values. Tumor protein P53 mutation correlated with lower TBR and higher ADC values. α-thalassemia/mental-retardation-syndrome-X-linked gene (ATRX) correlated with higher ADC values. 1p/19q codeletion and epidermal growth factor receptor (EGFR) mutations correlated with lower ADC values. Isocitrate dehydrogenase 1 (IDH1) mutations correlated with higher TBRmean values. No correlation existed between TBRmax/TBRmean/ADC/eADC values and phosphatase and tensin homolog mutations (PTEN) or O6-methylguanine-DNA methyltransferase (MGMT) promoter methylation. Furthermore, TBR/ADC combination had a higher diagnostic accuracy than each single imaging method for high-grade and IDH1-, hTERT-, and EGFR-mutated gliomas. This is the first study establishing the accurate diagnostic criteria for glioma based on FET-PET and DWI.

## Introduction

Gliomas are among the most common and severe primary intracranial tumors in humans, particularly glioblastoma (GBM) (WHO, grade IV). Newly diagnosed malignant gliomas are currently treated with surgical resection followed by radiotherapy and chemotherapy. However, despite treatment advancements, malignant glioma prognosis remains poor (1, 2). The main prognostic factors for glioma survival are extent resection, patient age and neurological performance (3). In addition, several molecular markers, including isocitrate dehydrogenase 1 (IDH1), epidermal growth factor receptor (EGFR), O6-methylguanine-DNA methyltransferase (MGMT) promoter methylation, telomerase reverse transcriptase (TERT), 1p/19q codeletion, phosphatase and tensin homolog (PTEN), and α-thalassemia/mental-retardation-syndrome-X-linked gene (ATRX), have been shown to be glioma prognostic factors (4–7), and their threshold values have been validated clinically. Moreover, some of these factors are used as a primary reference for post-operative therapy selection (8).

Currently, glioma diagnosis, grading, and molecular phenotyping mainly rely on postoperative histological examination, which requires obtaining a tumor sample using surgical resection or needle biopsy. In order to promote micro-invasive or non-invasive presurgical diagnosis, several studies have investigated the association of glioma molecular markers with specific tumoral imaging characteristics, including diffusion-weighted MRI (DWI), dynamic contrast-enhanced perfusion-weighted imaging (DCE-PWI), and magnetic resonance spectrometry (MRS) (9, 10). The increasing application of PET has improved the diagnosis and clinical management of gliomas (11, 12). Amino acid PET tracers, such as ^11^C-methyl-methionine (^11^C-MET), O-(2-^18^F-fluoroethyl)-L-tyrosine (^18^F-FET) and 3,4-dihydroxy-6-[^18^F]fluoro-L-phenylalanine (^18^F-DOPA), exhibit lower uptake in normal brain and inflammatory tissues than in gliomas and thus present clearer tumor borders with a higher tumor-to-background contrast than is achieved using 2-deoxy-2-[^18^F]fluoro-D-glucose (^18^F-FDG) (13). Previous reports indicated that FET uptake is associated with GBM genetic biomarkers. For example, IDH mutations were associated with higher methionine uptake on PET in patients with grade II–III gliomas (10, 14) compared with that in patients with high-grade glioma (HGG).

Glioma characterization using multi-modality imaging could have several clinical benefits (15, 16). Considering that DWI is an MR imaging modality that is based on measuring the random Brownian motion of water molecules within a given voxel, and apparent diffusion coefficient (ADC) has a comprehensible relation with tumor tissue characteristics (17, 18), the combination of DWI and FET can provide information about the amino-acid metabolism and water molecule motion in the tumor tissue, thus yielding more accurate and comprehensive molecular image analysis. Importantly, a combination of ADC and ^18^F-FET PET has been proven to detect glioma infiltration and phenotypes more accurately than standard MRI and other combination strategies such as ADC/cerebral blood flow (CBF) and FET/Flair (19).

Current reports regarding the correlation between tumor-to-background ratio (TBR)/ADC values and tumor type and grade are based on the average value calculated from the whole tumor or lesion. However, brain tumors, especially HGG, are known to exhibit intra-tumoral heterogeneity, with spatial differences in cellular phenotype and malignancy grade (20). Therefore, the analysis of the average TBR/ADC value of a whole tumor may not be truly representative to accurately determine the correlation with different brain tumor phenotypes. This could explain the wide ranges of previously reported TBR/ADC values for various tumor subtypes. We conceptualized that addressing this limitation could help improve the accuracy of this correlation.

Thus, the initial aim of this study was to eliminate the influence of glioma heterogeneity on the correlation between TBR/ADC values and tumor type and grade. As a first step in this direction, we retrospectively reviewed patients who underwent image-guided needle biopsies of the brain and had a preoperative T1, DWI, PET-MRI, and an intraoperative/early postoperative T1 for tracing the biopsy position and compared the findings with the histopathology report. Further, we studied the correlation between the molecular phenotypes of glioma and TBR/ADC values, which have never been validated by biopsy studies.

## Methods

### Patients

Eleven patients with newly diagnosed supratentorial gliomas who underwent hybrid ^18^F-FET-PET/MRI and DWI prior to biopsy from January 2019 to December 2019 were included in this study. Written informed consent was obtained from all the patients before PET/MR examinations. The Ethics Committee and Institutional Review Board of Xuanwu Hospital Capital Medical University approved this study. Patients were informed about the procedure and signed the consent forms.

### FET-PET/MRI

Patients had undergone the integrated PET/MRI within one week before the surgical procedure. To analyze the images, we used our previously published image-processing method (21). ^18^F-FET PET and MRI data were postprocessed and analyzed using PMOD version 3.505 (PMOD Ltd.). Different modalities were co-registered using nonaffine deformations and manually adjusted by referring to anatomic landmarks. The static PET images were resliced to the same voxel size as 3D T1 CEMRI with 1×1×1 mm for robust co-registration and more precise glioma volume calculations.

### Stereotactic Biopsy Procedures

The 11 patients underwent stereotactic biopsies within a week after MRI. The biopsies were performed under neuroimaging guidance using co-registered FET-PET and CE MR images loaded to the stereotactic navigation system Robotized Stereotactic Assistant (ROSA, Medtech) (22, 23). One to seven biopsy sites were selected per patient. Eloquent cortex areas and areas close to vesicular structures were excluded by both the experienced neurosurgeons (QT Lin and Y Cheng) and neuroradiologists (J Lu and SS Song). In all, 36 samples were obtained from lesions showing either contrast enhancement and increased FET uptake or increased FET uptake but no enhancement on CE MRI. No postoperative biopsy-related complications were observed.

### Intraoperative MRI and Image Fusion Verification

Intraoperative images were acquired using intraoperative MRI (Siemens, Verio) performed immediately after the biopsy procedure to verify the biopsy site, and 3D Slicer (Verson 4.1, SPL, Harvard Medical School) was used to register the preoperative MRI to the intraoperative MRI using the “General registration” module in the software. The biopsy site can be identified as a clear signal void in the intra/postoperative images, which was rigidly registered to the preoperative T1 and DWI. This was achieved using a module in 3D-Slicer called “Brains-fit”. However, this was a semi-automatic process, and a manual initialization of the registration was required. The “Transform” module was, therefore, used to manually register the images first, and then the “Generation registration (brain)” was applied to refine the registration.

### Sample Collection, Histological Grading, and Molecular Genetic Analysis

The diagnosis of glioma, according to the 2016 WHO classification criteria, was supported by the histopathological examination. Formalin-fixed paraffin-embedded (FFPE) tumor tissue blocks/sections or fresh tumor tissues were obtained from the hospitals, with confirmation of diagnosis and tumor purity provided by the pathologists. Grade II glioma was defined as low-grade glioma (LGG) and grade III-IV glioma was defined as high-grade glioma (HGG). Grade I glioma was not included in this study.

Genomic DNA from fresh tumor tissue and whole blood was extracted using the DNeasy Blood & Tissue Kit (Qiagen) according to the manufacturer’s protocols. FFPE samples were de-paraffinized with xylene followed by genomic DNA extraction using QIAamp DNA FFPE Tissue Kit (Qiagen) following the manufacturer’s instructions (23). Hybridization-based target enrichment was carried out with GeneseeqOne pancancer gene panel (416 cancer-relevant genes), and xGen Lockdown Hybridization and Wash Reagents Kit (Integrated DNA Technologies). The genetic markers most commonly used in glioma therapy and prognosis, namely, IDH1/2, 1p/19q, MGMT, hTERT, TP53, PTEN, EGFR, ATRX, were analyzed.

### Statistical Analysis

Statistical analysis was performed using SPSS version 22 (IBM). All quantitative data were presented as mean ± SD in the text. When comes to statistical analysis, we used a *Shapiro-Wilk* test and *Q-Q* plot to confirm normality for continuous variables. Then *Student’s t* test or the nonparametric *Wilcoxon test* were used to assess any statistically significant differences. (Detailed information in **Supplementary Table**). Descriptive statistics are presented as the mean and standard deviation or the median and range. A P value less than 0.05 was considered statistically significant. The accuracy of imaging combinations in tumor detection was determined using receiver operating characteristic (ROC) analysis. Using the imaging measurements as the diagnostic test and the histopathological analysis as the reference test, the areas under the ROC curve (AUCs) with 95% confidence intervals were calculated. AUCs of each single imaging method and the optimal imaging combination (TBRmean and ADC) were compared using a nonparametric analysis of clustered binary data to account for within-patient correlation.

## Results

**Figure 1:**
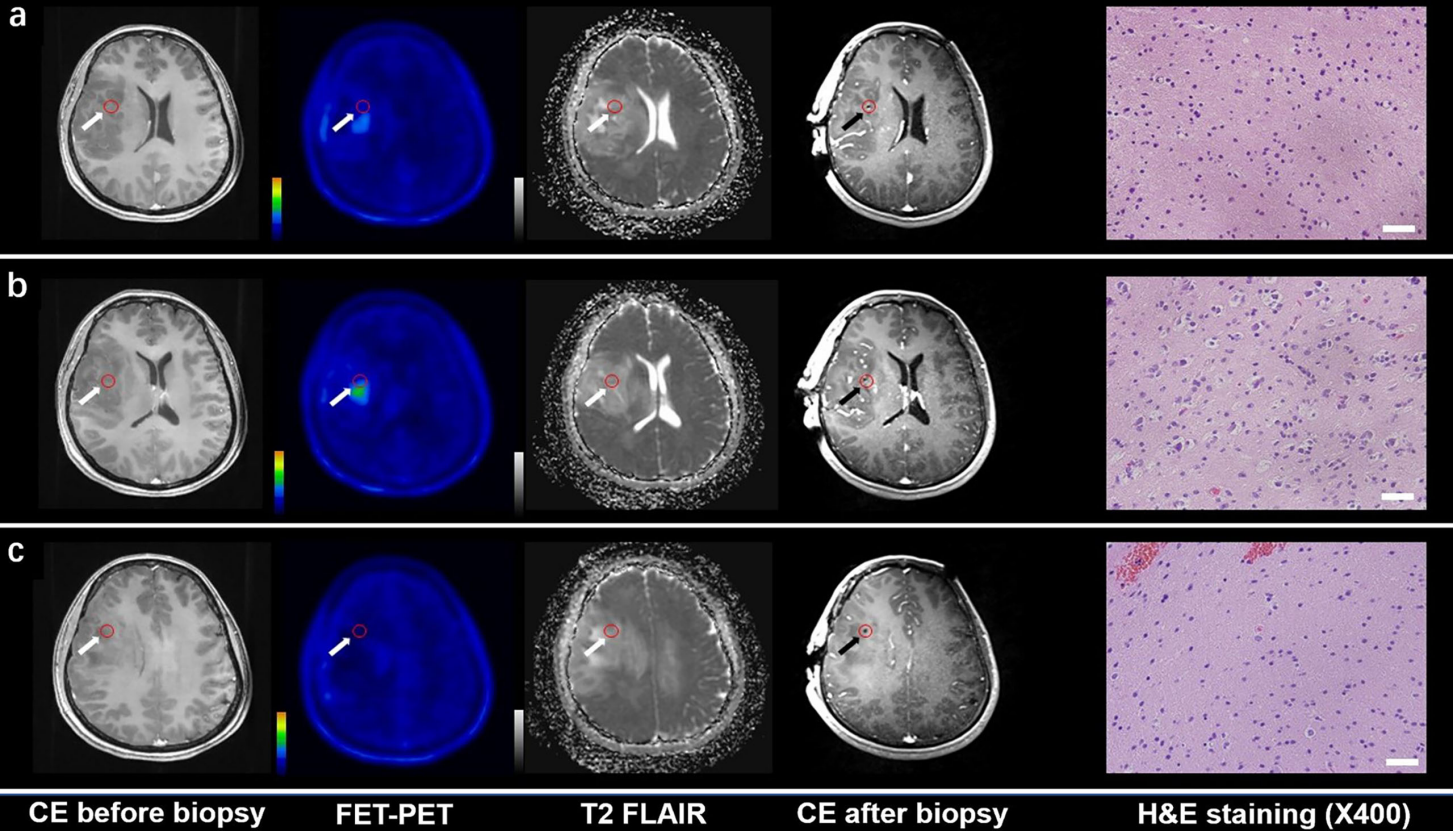
CE MRI, 18F-FET-PET and DWI-ADC map performed before biopsy, CE MRI performed after biopsy, and hematoxylin and eosin (H&E) staining (×40) of the biopsy samples. Samples located in different regions within glioma tissue with different FET-PET uptake and ADC value were taken. H&E staining showed WHO grade and the samples were also tested to analyze gene phenotypes. **(A, C)** Samples located in the region with increased FET-PET uptake and low ADC value in DWI. H&E staining showed this area contained reactive gliocyte proliferation without obvious atypia nuclear. **(B)** A sample located in a region with increased FET-PET uptake and high ADC value in DWI. H&E staining showed a cellular glioma corresponding to Oligodendroglioma of WHO grade II.

**Figure 2:**
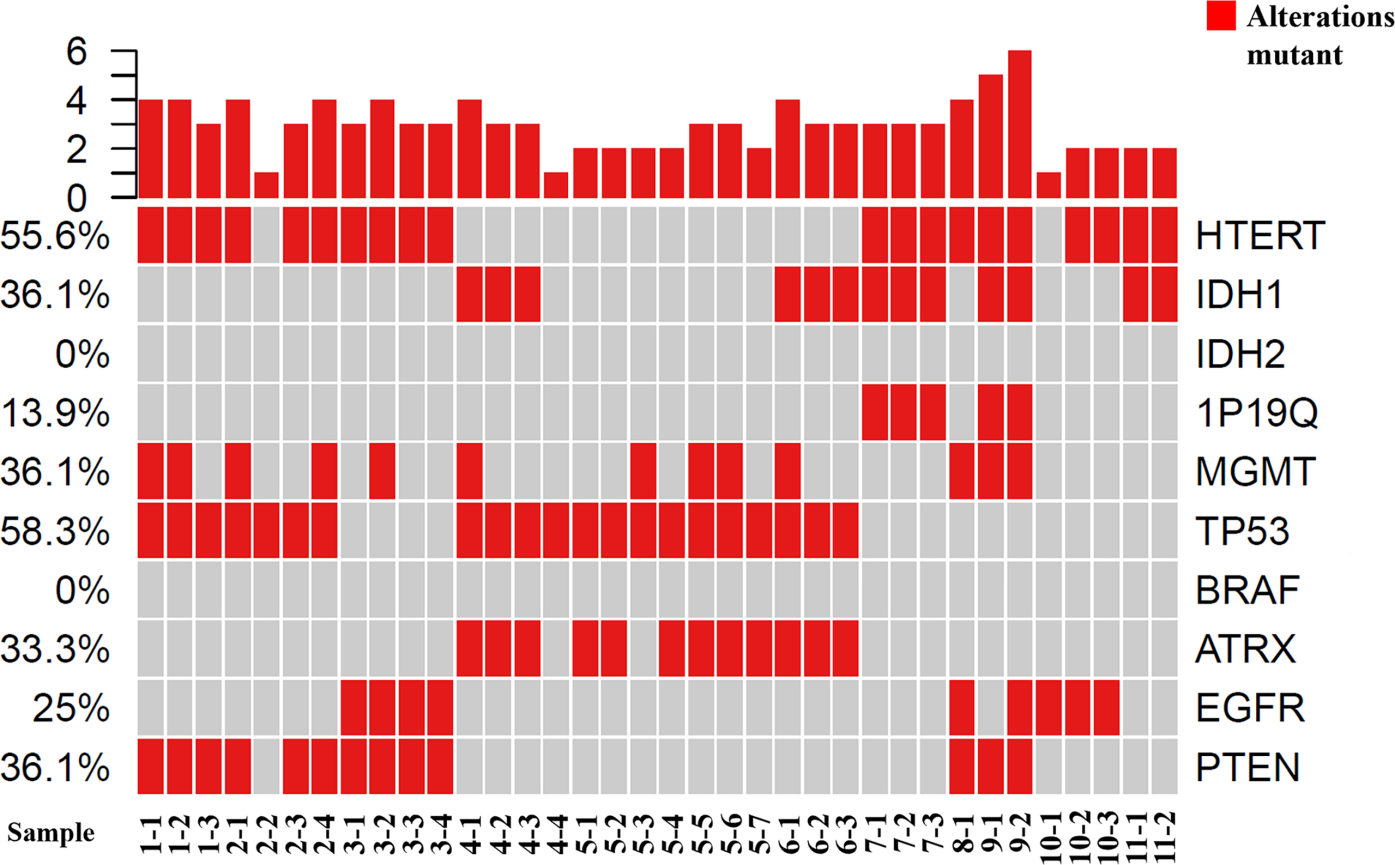
Tumor grading and molecular phenotypes in all of the samples using GeneseeqOne pancancer gene panel (Including IDH1/2, 1p/19q, MGMT, TP53, BRAF, ATRX, EGFR and PTEN).

**Figure 3:**
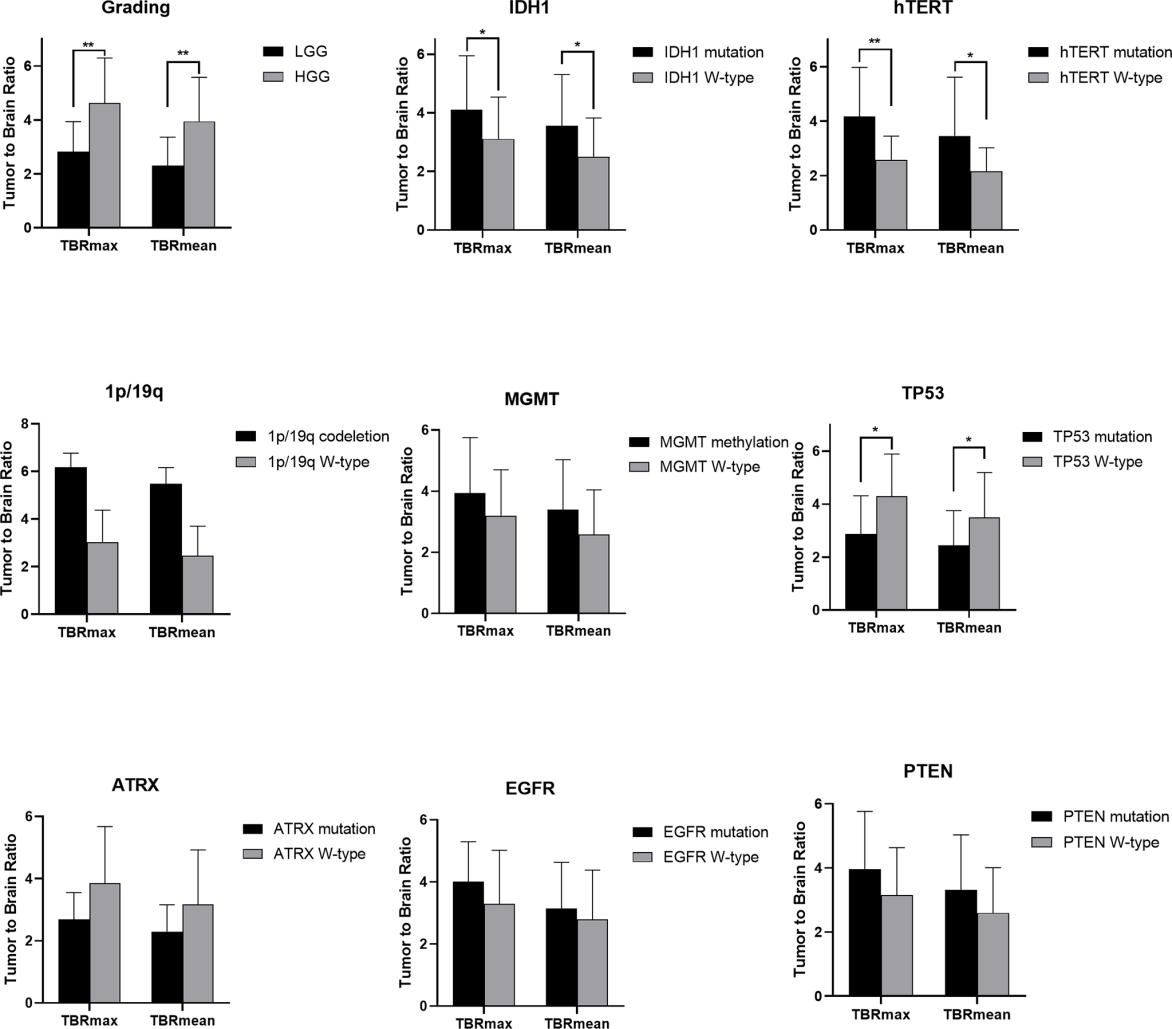
Statistic results of grading, IDH1/2, 1p/19q, MGMT, hTERT, TP53, PTEN, EGFR, ATRX mutations status and TBR (*P < 0.05, **P < 0.01).

**Figure 4:**
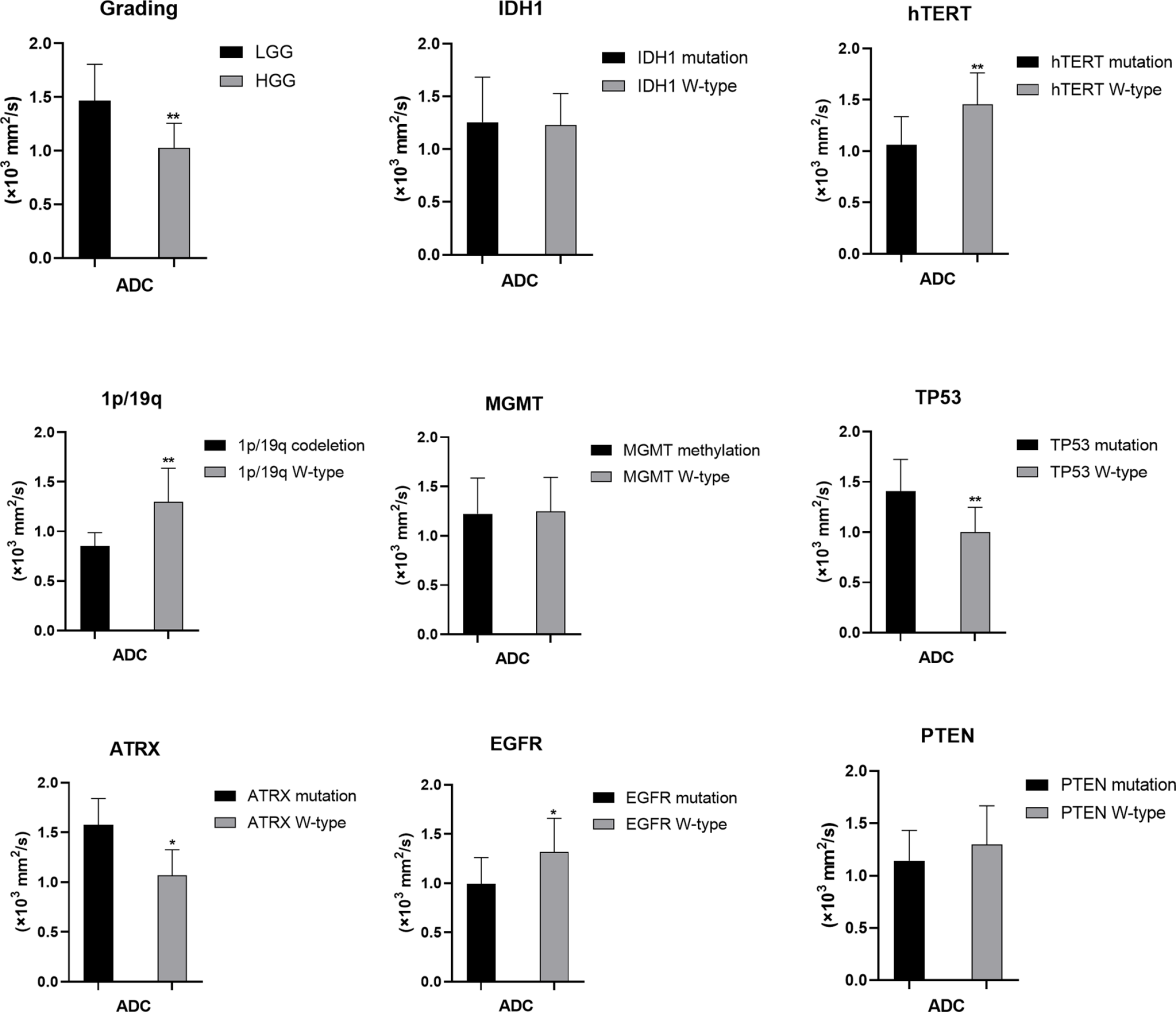
Statistic results of grading, IDH1/2, 1p/19q, MGMT, hTERT, TP53, PTEN, EGFR, ATRX mutations status and ADC (*P < 0.05, **P < 0.01).

**Figure 5:**
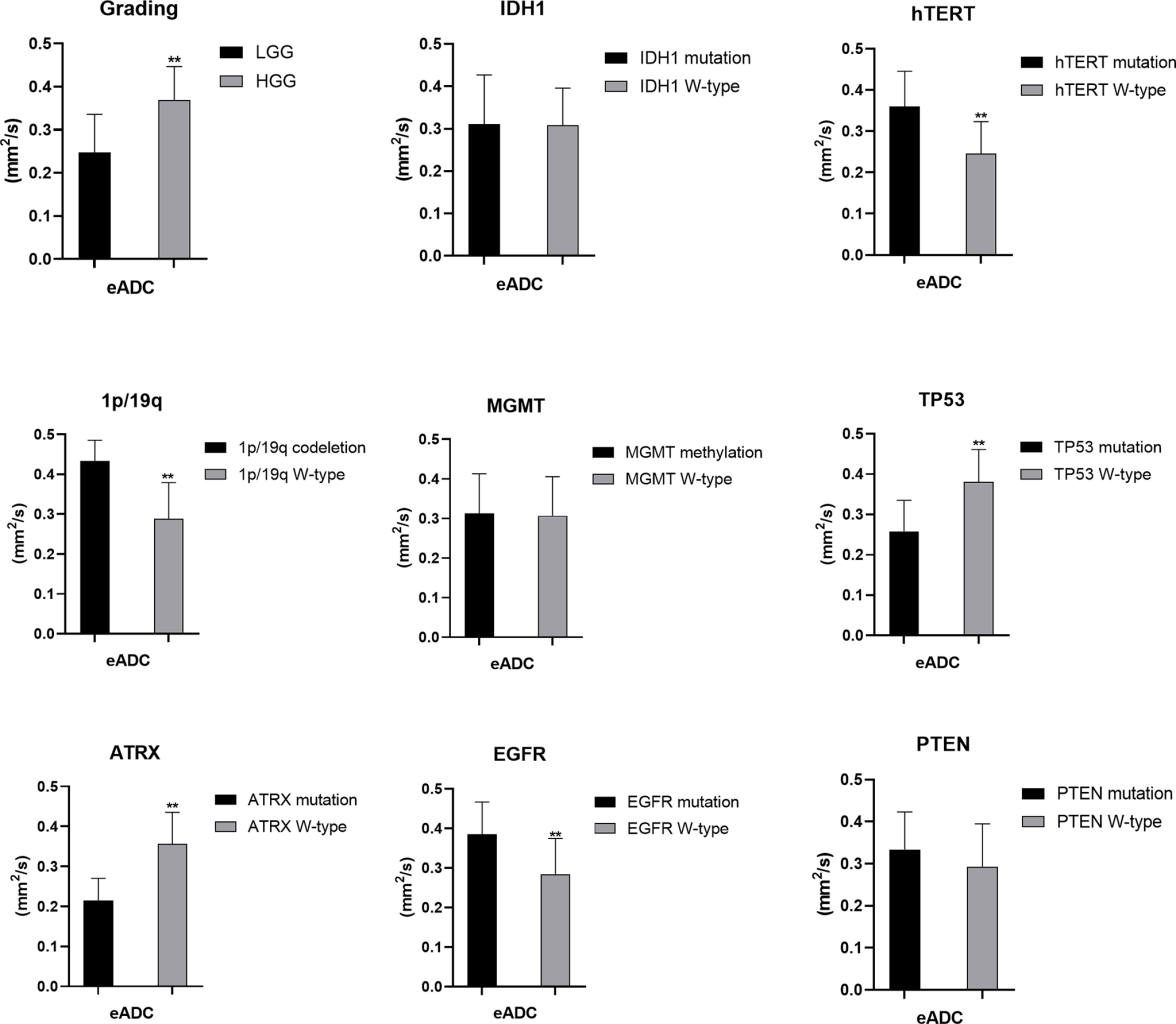
Statistic results of grading, IDH1/2, 1p/19q, MGMT, hTERT, TP53, PTEN, EGFR, ATRX mutations status and eADC (**P < 0.01).

Histopathology results of each biopsy site are listed in **Table 1**. The biopsy samples were heterogeneous both histologically and molecularly in some patients as different pathological results were described in the biopsy reports even in the same patient (**Figure 1**). The molecular phenotypes of the samples, as determined using the GeneseeqOne pancancer gene panel, are listed in **Figure 2**. The correlations of the mutation status of the genetic markers with TBR and ADC/eADC are shown in **Figures 3**–**5**, respectively. Our initial screening results revealed no relation between TBR values and MGMT promoter methylation and ATRX, EGFR, and PTEN mutations. Moreover, ADC values cannot predict the mutation status of IDH and PTEN or MGMT promoter methylation.

**Table 1:** Histopathology results of each biopsy site and patient.

Sample	Patient	Histopathology reports	WHO Grade (Sample)	WHO Grade (Patient)
1	1	Anaplastic astrocytoma	III	IV
2		Glioblastoma	IV	
3		Anaplastic astrocytoma	III	
4	2	Glioblastoma	IV	IV
5		Gliocyte proliferation	–	
6		Normal tissue + LGG	II	
7		Glioblastoma	IV	
8	3	Glioblastoma	IV	IV
9		Glioblastoma + necrosis	IV	
10		Gliocyte proliferation + tumor cells infiltration	–	
11		Normal tissue + LGG	II	
12	4	Normal tissue + astrocytoma	II	II
13		LGG + hemorrhage	II	
14		LGG + hemorrhage	II	
15		Gliocyte proliferation + tumor cells infiltration	–	
16	5	Astrocytoma	II	III
17		Normal tissue + astrocytoma	II	
18		Normal tissue + LGG	II	
19		Normal tissue + LGG	II	
20		Normal tissue + LGG	II	
21		Astrocytoma + Anaplastic astrocytoma	III	
22		Normal tissue + LGG	II	
23	6	Oligodendroglioma	II	II
24		Oligodendroglioma	II	
25		Normal tissue + LGG	II	
26	7	Anaplastic oligodendroglioma	III	III
27		Anaplastic oligodendroglioma	III	
28		Oligodendroglioma	II	
29	8	Glioblastoma	IV	IV
30	9	Anaplastic oligodendroglioma	III	III
31		Anaplastic oligodendroglioma	III	
32	10	Gliocyte proliferation + tumor cells infiltration	–	III
33		Oligodendroglioma	II	
34		Astrocytoma + Anaplastic	III	
35	11	Gliocyte proliferation + tumor cells infiltration	–	II
36		Astrocytoma	II	

### FET Uptake and ADC Values Predict Glioma Grading

For tumor grading, the potential for feature-based differentiation between WHO II-IV gliomas was assessed. The TBRmax values were 2.823 ± 1.112 vs. 4.624 ± 1.675 in LGG and HGG (p<0.05), respectively, whereas the TBRmean values were 2.305 ± 1.056 vs. 3.949 ± 1.630 (p<0.05) in LGG and HGG, respectively. The mean ADC values were 1.465 ± 0.341 vs. 1.024 ± 0.232 (×10^3^ mm^2^/s) in LGG and HGG (p<0.05), while the mean eADC values were 0.247 ± 0.089 vs. 0.369 ± 0.078 (mm^2^/s) in LGG and HGG (p<0.05), respectively (**Supplementary Table 1**).

### FET Uptake Levels Are Influenced by the Status of hTERT, IDH1, and TP53 Mutations and 1p/19q Codeletion

The mutation status of hTERT, 1p/19q, IDH1 and TP53 was predicted based on FET uptake in terms of TBRmax and TBRmean. Samples with IDH1 mutations showed higher TBRmean values than those of samples with wild-type IDH1 (3.552 ± 1.752 vs. 2.498 ± 1.322, p<0.05) (**Supplementary Table 2**). Higher TBRmax and TBRmean values were found in samples with hTERT mutation (4.173 ± 1.803 vs. 2.584 ± 0.866 and 3.455 ± 1.766 vs. 2.158 ± 0.864, separately, p<0.05) compared with samples with wild-type gene (**Supplementary Tables 3, 4**). Lower TBRmax and TBRmean values were found in samples with TP53 mutation compared with those in samples with the wild-type gene (2.872 ± 1.442 vs. 4.300 ± 1.590 and 2.441 ± 1.312 vs. 3.492 ± 1.702, p<0.05) (**Supplementary Table 6**). Furthermore, the FET uptake level was not correlated with the mutation status of ATRX, EGFR, PTEN, 1p/19q codeletion and MGMT methylation as p>0.05 (*Student’s T-test or Wilcoxon test*).

### ADC Values Predict the Mutation Status of hTERT, TP53, EGFR, and ATRX and 1p/19q Codeletion

The mutation status of hTERT and TP53 and 1p/19q codeletion were predicted by ADC values. Higher mean ADC values were found in samples with TP53 (1.406 ± 0.318 vs. 1.000 ± 0.247, p<0.05) and ATRX mutations (1.576 ± 0.265 vs. 1.068 ± 0.257, p<0.05) (**Supplementary Tables 6, 7**), whereas lower values were found in samples with hTERT and EGFR mutations (1.062 ± 0.274 vs. 1.456 ± 0.307, p<0.05) and 1p/19q codeletion (0.852 ± 0.136 vs. 1.299 ± 0.338, p<0.05) (**Supplementary Tables 3, 4, 8**). These findings were statistically significant (p<0.05, Student’s T-test). ADC values could not predict the mutation status of IDH and PTEN and MGMT promoter methylation (p>0.05) (*Student’s T-test or Wilcoxon test*).

### eADC Values Predic the Mutation Status of hTERT, TP53, EGFR, and ATRX and 1p/19q Codeletion

The mutation status of the hTERT, 1p/19q and TP53 was predicted by eADC values. Higher mean eADC values were found in samples with hTERT (0.360 ± 0.085 vs. 0.246 ± 0.077, p<0.05) and EGFR mutation (0.385 ± 0.082 vs. 0.284 ± 0.091, p<0.05) and 1p/19q codeletion (0.433 ± 0.052 vs. 0.289 ± 0.090) (**Supplementary Tables 3, 4, 8**), whereas lower values were found in samples with TP53 (0.258 ± 0.077 vs. 0.381 ± 0.080, p<0.05) and ATRX mutation (0.215 ± 0.055 vs. 0.356 ± 0.079, p<0.05) (**Supplementary Tables 6, 7**). These findings were statistically significant (p<0.05, Student’s T-test). However, eADC values could not predict the mutation status of IDH and PTEN and MGMT promoter methylation (P>0.05). (*Student’s T-test or Wilcoxon test*).

### Comparison of the Diagnostic Accuracy of Single- and Multi-Modality Imaging

**Figure 6:**
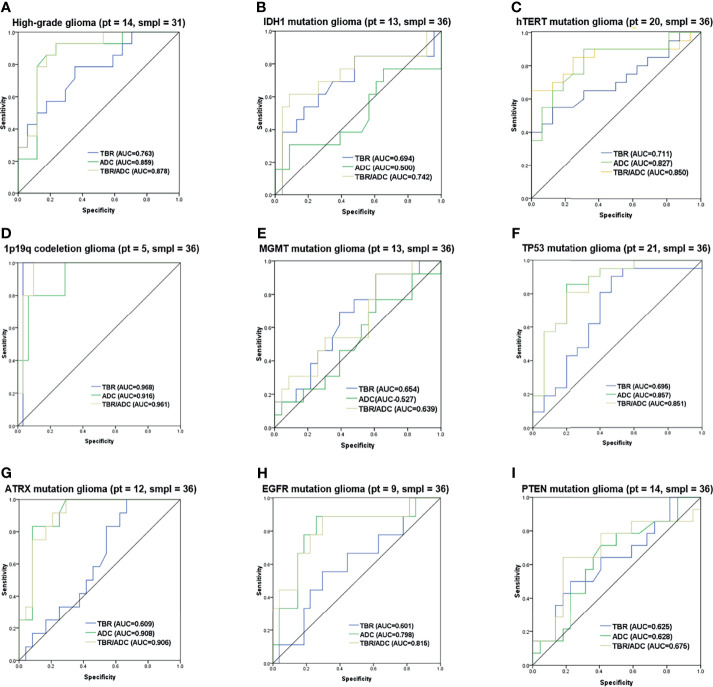
Receiver operating characteristic curves of 18F-FET PET, and DWI imaging combination. ROC curves with the AUC of the optimal imaging combinations in yellow, FET-PET in blue and DWI in green in cyan for **(A)** grading, **(B)** IDH1 mutation, **(C)** hTERT mutation, **(D)** 1p/19q codeletion, **(E)** MGMT mutation, **(F)** TP53 mutation, **(G)** ATRX mutation, **(H)** EGFR mutation and **(I)** PTEN mutation. The number of patients and samples of each ROC analysis is displayed in the title.

The diagnostic accuracy of the FET/ADC combination was significantly higher than that of each single imaging method with respect to both glioma grading and molecular phenotyping. Moreover, the AUCs revealed that the accuracy of the combination was significantly higher than that of single ADC or ^18^F-FET PET (**Figure 6A**). In the gene-specific subgroup analysis, the diagnostic accuracy of combined TBR/ADC was significantly higher than that of each imaging mode for gliomas with IDH1 mutation (**Figure 6B**; AUC, 0.742 vs. 0.690 [TBRmean] and 0.500 [ADC]), so as for hTERT (**Figure 6C**; AUC, 0.850 vs. 0.711 [TBRmean] and 0.827 [ADC]) and EGFR mutations (**Figure 6H**; AUC, 0.815 vs. 0.601 [TBRmean] and 0.798 [ADC]). By contrast, the diagnostic accuracy of TBR/ADC was not significantly higher than that of single ADC or ^18^F-FET PET for gliomas with 1p/19q codeletion, MGMT promoter methylation and PTEN mutation (**Figures 6D, E, I**). The diagnostic accuracies of TBR/ADC and ADC were almost identical for gliomas with TP53 and ATRX mutation (**Figures 6F, G**).

## Discussion

The decision for surgical resection of a tumor depends on the possibilities of maximal function retention and minimal recurrence. For example, when the gadolinium-enhanced portion of the glioma is completely resected, 90% of recurrences occur at the margin of the surgical resection in the macroscopically normal peritumoral brain zone. However, as the patient may lose important brain function owing to over-resection, the pre-surgical plan needs to be comprehensive. Multi-modal MRI is widely used for brain tumor diagnosis. Advanced imaging techniques, including perfusion and diffusion MRI, as well as PET, are being actively investigated to overcome some of the limitations of cMRI modalities (24). Furthermore, the multi-model MRI has been introduced to analyze the heterogeneity of GBM, and the use of the ADC value is widely accepted in the evaluation of the heterogeneity of GBM (25). During the last few years, interest has increased in the development of DWI ADC and FET-PET applications for tumor grading, molecular subtyping, and the assessment of treatment response.

The combination of ADC and FET-PET imaging has been proven to be more accurate than standard MRI in the detection of infiltration in enhancing gliomas (26). However, there are no reports of imaging combinations of FET-PET and ADC for molecular phenotyping of gliomas. In the current study, we calculated the value of image parameters (TBR/ADC) in the needle biopsy sites to provide a more accurate TBR/ADC value for tumor tissues of different grades and molecular phenotypes. Since previous studies had reported the value and feasibility of hybrid PET/MR-guided brain biopsies (27–29). Our study aims to further assess the mutation status of gliomas using hybrid PET/MRI in combination with stereotactic biopsy to eliminate the influence of heterogeneity.

Consistent with previous histopathological reports (30), we observed that the glioma histopathological grade was positively correlated with TBRmax and TBRmean and negatively correlated with ADC values. Furthermore, as in a previous study (31), we validated the findings using needle biopsy to eliminate the effects of glioma heterogeneity. In addition, IDH mutation and 1p/19q codeletion are important markers for both glioma typing and prognosis (32), as well as for differentiating oligodendrogliomas from GBMs and astrocytomas (33). Based on the 2016 WHO classification, oligodendrogliomas without a 1p/19q codeletion are classified as astrocytomas (32). A number of studies have investigated the potential of exclusive MRI or PET imaging to predict IDH mutation (32), suggesting potential differences between the uptake patterns of different amino acid PET tracers in gliomas due to the specific metabolic profiles of IDH-mutated gliomas. However, previously reported correlations between IDH mutation status and FET uptake varied due to the differences in the amino acid PET tracers and calculation methods (32). The results of our point-to-point biopsy method indicated a higher [^18^F]-FET uptake in only IDH-mutated gliomas. This was consistent with the findings of Verger who used [^18^F]-FDOPA PET to predict the IDH mutation status (34). In our study, low ADC values were found in the 1p/19q codeletion group. Thus, the combination of FET-PET and DWI has the potential to preoperatively differentiate astrocytomas from oligodendroglioma. None IDH2 mutation was found in our samples. Histological and molecular information obtained using multi-modality imaging including advanced MRI imaging may help the surgeon prepare a better surgical plan for tumor resection and needle biopsy than that prepared based on conventional MRI imaging, which only provides structural tumor information (35, 36).

For glioma phenotyping, IDH1/2, 1p/19q, MGMT, hTERT, TP53, PTEN, EGFR, and ATRX mutations are usually regarded as the key factors influencing postoperative therapeutic decisions, such as radiotherapy, chemotherapy, molecular targeted therapy and immune therapy (37). This is the first study to report a correlation between hTERT mutation status and TBRmax and TBRmean. This finding is in contrast to those of a previous study that reported that these factors did not have any significant correlations (8). These contrasting results can be attributed to the differences in the methodologies of the two studies. However, our results are more precise and accurate because we determined the correlations based on examination of biopsy sites. Further, lower ADC and higher eADC values could preoperatively predict the presence of hTERT mutation. The hTERT mutation has been specifically found in HGGs, especially in GBM; therefore, hTERT could be a promising therapeutic target in HGG, especially recurrent HGG (38). Furthermore, our study is the first to find the value of ADC in predicting TP53 and EGFR, which are also relevant molecular targets (39). Therefore, our results have the potential to provide precise molecular information based on image, so as to be a reference for individualized treatment plan of molecular targeted therapy and radiotherapy.

Our study also detected two additional molecular biomarkers, PTEN and ATRX. ATRX mutations have been observed in 71% of grade II-III astrocytoma, 68% of oligoastrocytoma, and 57% of secondary GBM. Loss of ATRX is associated with improved progression-free and overall survival (40). ATRX can be used not only in the molecular classification of gliomas but also as a new glioma therapeutic target (41). Our study is the first to report a relationship between ATRX and the imaging parameters PET/TBR and DWI/ADC. A higher ADC value was specifically observed in ATRX-mutated gliomas. Furthermore, PTEN is the most frequently altered tumor suppressor gene in GBM, and its loss or mutation has been implicated in the resistance to therapies, such as tyrosine kinase inhibitors, due to permissive activation of the AKT pathway. However, depletion of PTEN has also been shown to sensitize tumor cells to therapies that rely on DNA damage, such as ionizing radiation (42). We did not find a correlation between 18F-FET uptake, ADC value, and PTEN mutation status was observed.

There are no previous studies using imaging modality combinations for investigating mutations in MGMT, hTERT, TP53, PTEN, EGFR, and ATRX in gliomas. Thus, the present findings can be an initial step of exploring precise molecular imaging of glioma. A recent study, reported that a combination of ADC and FET-PET detected infiltration in enhancing gliomas, as well as in HGG and oligodendroglioma, more accurately than standard MRI and FET-PET (19). However, our study is the first to perform glioma molecular phenotyping using a TBR/ADC combination strategy. Moreover, our findings were validated by hybrid PET/MR-guided biopsies. Interestingly, the combination of TBR/ADC had a significantly higher diagnostic accuracy than that of each single imaging method in both glioma grading and predicting the mutation status of IDH1, hTERT, and EGFR. When comes to the prediction of glioma grading and above three genes, combined modality of PET and DWI are commended.

The present study is limited by the relatively small number of patients in the subgroup analyses, especially in the case of gliomas with 1p/19q codeletion and IDH2 mutation. However, our data provide initial evidence for the correlation between imaging parameters and molecular phenotypes. In addition to IDH1/2, 1p/19q, MGMT, hTERT, TP53, PTEN, EGFR, and ATRX, which were investigated here, we plan to evaluate other genetic markers related to glioma characteristics and prognosis, as well as those recognized as therapeutic and immune targets.

## Conclusion

TBR/ADC values acquired using PET-MRI and DWI could be useful diagnostic tools for radiologists for better preoperative understanding of tumor characteristics and could also guide surgeons in pre-surgical planning and treatment decision making. While further research is required, we believe our method of using the TBR/ADC values from the biopsy site provides better representation of the actual tumor pathology.

## Data Availability Statement

The raw data supporting the conclusions of this article will be made available by the authors, without undue reservation.

## Ethics Statement

Written informed consent was obtained from the individual(s) for the publication of any potentially identifiable images or data included in this article.

## Author Contributions

All authors contributed to the study conception and design. Material preparation was by YC, SS, YW, GX, YA, and LW. Data collection, and analysis were performed by JM, HY, ZQ, XX, JB, LX, ZH, and TS. The first draft of the manuscript was written by YC, SS, LW, JL, and QL. All authors contributed to the article and approved the submitted version.

## Funding

This work was funded by the Youth Program of National Natural Science Foundation of China (81802485 to YC), Beijing New-star Plan of Science and Technology (Z201100006820148 to YC). Beijing Municipal Administration of Hospitals’ Ascent Plan (DFL20180802 to JL).

## Conflict of Interest

Author TS was employed by Nanjing Geneseeq Technology Inc., Nanjing, China.

The remaining authors declare that the research was conducted in the absence of any commercial or financial relationships that could be construed as a potential conflict of interest.

## Publisher’s Note

All claims expressed in this article are solely those of the authors and do not necessarily represent those of their affiliated organizations, or those of the publisher, the editors and the reviewers. Any product that may be evaluated in this article, or claim that may be made by its manufacturer, is not guaranteed or endorsed by the publisher.
