# Supplementary material for: Glioma Imaging by O-(2-18F-Fluoroethyl)-L-Tyrosine PET and Diffusion-Weighted MRI and Correlation With Molecular Phenotypes, Validated by PET/MR-Guided Biopsies
Source: Front Oncol. 2021 Nov 29;11:743655. doi: 10.3389/fonc.2021.743655 (PMC8666958; doi:10.3389/fonc.2021.743655)
Supplement: Supplementary file 1 [file Table_1.docx]

**Supplementary Table 1. Relationship between glioma grade and TBR/ADC (mean ± SD).**

|  | FET-PET | | DWI | |
| --- | --- | --- | --- | --- |
|  | TBRmax | TBRmean | ADC (×10^3^ mm^2^/s) | eADC (mm^2^/s) |
| LGG (n=17) | 2.823±1.112 | 2.305±1.056 | 1.465±0.341 | 0.247±0.089 |
| HGG (n=14) | 4.624±1.675 | 3.949±1.630 | 1.024±0.232 | 0.369±0.078 |
| p | 0.0016 | 0.0027 | 0.0004 | 0.0005 |

“*Student-t test*” was used to compare FET-PET and DWI in LGG and HGG

**Supplementary Table 2. Relationship between IDH1 mutation and TBR/ADC (mean±SD)**

|  | FET-PET | | DWI | |
| --- | --- | --- | --- | --- |
|  | TBRmax | TBRmean | ADC (×10^3^ mm^2^/s) | eADC (mm^2^/s) |
| Mutation (n=13) | 4.104±1.843 | 3.552±1.752 | 1.256±0.428 | 0.311±0.116 |
| W-type(n=23) | 3.107±1.430 | 2.498±1.322 | 1.227±0.302 | 0.308±0.088 |
| p | 0.033**^#^** | 0.046**^#^** | 0.8190* | 0.9278* |

#: “*Wilcoxon test*” was used to compare the values of TBRmax and TBRmean in IDH1 mutation group and wild-type group since *Shapiro-Wilk* test indicated non-normally distributed data.

*: “*Student t test*” was used to compare the values of ADC and eADC in IDH1 mutation group and wild-type group since *Shapiro-Wilk* test indicated normally distributed data.

**Supplementary Table 3. Relationship between hTERT mutation and TBR/ADC (mean±SD)**

|  | FET-PET | | DWI | |
| --- | --- | --- | --- | --- |
|  | TBRmax | TBRmean | ADC (×10^3^ mm^2^/s) | eADC (mm^2^/s) |
| Mutation (n=20) | 4.173±1.803 | 3.455±1.766 | 1.062±0.274 | 0.360±0.085 |
| W-type(n=16) | 2.584±0.866 | 2.158±0.864 | 1.456±0.307 | 0.246±0.077 |
| p | 0.0034* | 0.0132* | 0.007^#^ | 0.0003* |

#: “*Wilcoxon test*” was used to compare the values of ADC in hTERT mutation group and wild-type group since *Shapiro-Wilk* test indicated non-normally distributed data.

*: “*Student t test*” was used to compare the values of TBRmax, TBRmax and eADC in hTERT mutation group and wild-type group since *Shapiro-Wilk* test indicated normally distributed data.

**Supplementary Table 4. Relationship between 1p/19q codeletion and TBR/ADC (mean±SD)**

|  | FET-PET | | DWI | |
| --- | --- | --- | --- | --- |
|  | TBRmax | TBRmean | ADC (×10^3^ mm^2^/s) | eADC (mm^2^/s) |
| Mutation(n=5) | 6.164±0.601 | 5.480±0.679 | 0.852±0.136 | 0.433±0.052 |
| W-type(n=31) | 3.032±1.336 | 2.459±1.240 | 1.299±0.338 | 0.289±0.090 |
| p | 0.08^#^ | 0.08^#^ | 0.00753* | 0.00176* |

#: “*Wilcoxon test*” was used to compare the values of TBRmax and TBRmean in 1p/19q codeletion group and wild-type group since *Shapiro-Wilk* test indicated non-normally distributed data.

*: “*Student t test*” was used to compare the values of ADC and eADC in 1p/19q codeletion group and wild-type group since *Shapiro-Wilk* test indicated normally distributed data.

**Supplementary Table 5. Relationship between MGMT** **methylation** **and TBR/ADC (mean±SD)**

|  | FET-PET | | DWI | |
| --- | --- | --- | --- | --- |
|  | TBRmax | TBRmean | ADC (×10^3^ mm^2^/s) | eADC (mm^2^/s) |
| Mutation(n=13) | 3.944±1.812 | 3.398±1.636 | 1.222±0.364 | 0.313±0.100 |
| W-type(n=23) | 3.197±1.505 | 2.586±1.460 | 1.245±0.347 | 0.307±0.0987 |
| p | 0.2061* | 0.1453* | 0.8567* | 0.8770* |

*: “*Student t test*” was used to compare the values of TBRmax, TBRmean, ADC and eADC in MGMT methylation group and wild-type group since *Shapiro-Wilk* test indicated normally distributed data.

**Supplementary Table 6. Relationship between TP53 mutation and TBR/ADC (mean±SD)**

|  | FET-PET | | DWI | |
| --- | --- | --- | --- | --- |
|  | TBRmax | TBRmean | ADC (×10^3^ mm^2^/s) | eADC (mm^2^/s) |
| Mutation(n=21) | 2.872±1.442 | 2.441±1.312 | 1.406±0.318 | 0.258±0.077 |
| W-type(n=15) | 4.300±1.590 | 3.492±1.702 | 1.000±0.247 | 0.381±0.080 |
| p | 0.027**^#^** | 0.047**^#^** | 0.0003* | 0.0000753* |

#: “*Wilcoxon test*” was used to compare the values of TBRmax and TBRmean in TP53 mutation group and wild-type group since *Shapiro-Wilk* test indicated non-normally distributed data.

*: “*Student t test*” was used to compare the values of ADC and eADC in TP53 mutation group and wild-type group since *Shapiro-Wilk* test indicated normally distributed data.

**Supplementary Table 7. Relationship between ATRX mutation and TBR/ADC (mean±SD)**

|  | FET-PET | | DWI | |
| --- | --- | --- | --- | --- |
|  | TBRmax | TBRmean | ADC (×10^3^ mm^2^/s) | eADC (mm^2^/s) |
| Mutation(n=12) | 2.694±0.856 | 2.296±0.868 | 1.576±0.265 | 0.215±0.055 |
| W-type(n=24) | 3.853±1.825 | 3.170±1.757 | 1.068±0.257 | 0.356±0.079 |
| p | 0.0504* | 0.1230* | 0.015^#^ | 0.000006468* |

#: “*Wilcoxon test*” was used to compare the values of ADC in ATRX mutation group and wild-type group since *Shapiro-Wilk* test indicated non-normally distributed data.

*: “*Student t test*” was used to compare the values of TBRmax, TBRmax and eADC in ATRX mutation group and wild-type group since *Shapiro-Wilk* test indicated normally distributed data.

**Supplementary Table 8. Relationship between EGFR mutation and TBR/ADC (mean±SD)**

|  | FET-PET | | DWI | |
| --- | --- | --- | --- | --- |
|  | TBRmax | TBRmean | ADC (×10^3^ mm^2^/s) | eADC (mm^2^/s) |
| Mutation(n=9) | 4.007±1.287 | 3.135±1.500 | 0.993±0.267 | 0.385±0.082 |
| W-type(n=27) | 3.286±1.732 | 2.793±1.590 | 1.318±0.341 | 0.284±0.091 |
| p | 0.26^#^ | 0.767^#^ | 0.01578* | 0.00721* |

#: “*Wilcoxon test*” was used to compare the values of TBRmax and TBRmean in EGFR mutation group and wild-type group since *Shapiro-Wilk* test indicated non-normally distributed data.

*: “*Student t test*” was used to compare the values of ADC and eADC in EGFR mutation group and wild-type group since *Shapiro-Wilk* test indicated normally distributed data.

**Supplementary Table 9. Relationship between PTEN mutation and TBR/ADC (mean±SD)**

|  | FET-PET | | DWI | |
| --- | --- | --- | --- | --- |
|  | TBRmax | TBRmean | ADC (×10^3^ mm^2^/s) | eADC (mm^2^/s) |
| Mutation(n=13) | 3.969±1.793 | 3.320±1.713 | 1.142±0.290 | 0.334±0.089 |
| W-type(n=23) | 3.147±1.487 | 2.598±1.410 | 1.297±0.371 | 0.293±0.102 |
| p | 0.1566 | 0.1899 | 0.2068 | 0.2455 |

“*Student t test*” was used to compare the values of TBRmax, TBRmean, ADC and eADC in PTEN mutation group and wild-type group since *Shapiro-Wilk* test indicated normally distributed data.
